# Diabetes status, fasting glucose and HbA1c, and outcomes after myocardial infarction: a retrospective registry-based Swedish cohort study

**DOI:** 10.1136/bmjopen-2026-121970

**Published:** 2026-09-18

**Authors:** Azzum Haider, Emil Hagström, Divan Ishak, Nermin Hadziosmanovic, Anna Norhammar, Matthijs Velders, Gorav Batra

**Affiliations:** 1 Uppsala Universitet, Uppsala, Sweden; 2 Cardiology, Västerås Central Hospital, Västerås, Sweden; 3 Department of Medical Sciences, Cardiology, Uppsala University, Uppsala, Sweden; 4 Uppsala Clinical Research Centre, Uppsala University, Uppsala, Sweden; 5 Cardiology, Uppsala University, Uppsala, Sweden; 6 Karolinska Institutet, Stockholm, Sweden; 7 Capio Sankt Görans Sjukhus, Stockholm, Sweden; 8 Department of Clinical Physiology, Västerås Hospital, Västerås, Sweden

**Keywords:** DIABETES & ENDOCRINOLOGY, Myocardial infarction, Cardiovascular Disease, Ischaemic heart disease

## Abstract

**Abstract:**

**Objectives:**

This study aimed to assess cardiovascular (CV) risk across the full spectrum of dysglycaemia levels following myocardial infarction (MI).

**Design:**

Retrospective registry-based cohort study.

**Setting:**

All patients with MI in Sweden registered in the cardiac rehabilitation registry within the Swedish Web-system for Enhancement and Development of Evidence-based care in Heart disease Evaluated According to Recommended Therapies (SWEDEHEART) between 2006 and 2017.

**Participants:**

In total, 47 558 patients over the age of 18 attending cardiac rehabilitation post-MI, with available information on glycaemic levels.

**Primary and secondary outcome measures:**

The primary outcome was major adverse CV events (MACE), a composite of all-cause mortality, MI and ischaemic stroke until the end of follow-up (30 June 2018). Secondary outcomes included all the components of MACE, CV mortality and hospitalisation for heart failure.

**Results:**

Pre-diabetes and possible diabetes were present in 20.6%, new-onset diabetes in 3.2% and known diabetes in 21.1%. During a median follow-up of 4.7 years, 9321 patients experienced MACE. A stepwise increase across glycaemic categories was observed, with HRs of 1.07 (95% CI 1.00 to 1.14) for pre-diabetes, 1.22 (95% CI 1.11 to 1.34) for possible diabetes, 1.22 (95% CI 1.08 to 1.37) for new-onset diabetes and 1.73 (95% CI 1.64 to 1.82) for known diabetes, compared with normoglycaemia. A near-linear association between fasting glucose and outcomes was observed across all patients, while glycated haemoglobin appears predictive mainly in those with established diabetes.

**Conclusions:**

Glycaemic disturbances early after MI, including hyperglycaemia below the diagnostic threshold for diabetes, are associated with a progressively higher risk of adverse outcomes. Beyond routine screening, these findings support close follow-up of at-risk patients, irrespective of diabetes status.

**Strengths and limitations of this study**

- Large nationwide cohort study with consecutive enrolment in a mandatory reliable data source.
- Use of the cardiac rehabilitation visit as the index time point provides a clinically stable and standardised assessment of glycaemic status and cardiovascular risk factors, reducing misclassification related to acute stress hyperglycaemia and improving comparability across patients.
- Associations with dysglycaemia at lower thresholds as considered for diabetes, showing a relationship with high major adverse cardiovascular events and paving the way for early identification and risk stratification.
- Observational data are limited and prone to residual confounding.
- Fasting glucose and glycated haemoglobin were measured at a single post-myocardial infarction time point and may not reflect long-term glycaemic exposure or variability, leading to potential misclassification.

## Introduction

 Patients with type 2 diabetes are at increased lifetime risk of cardiovascular (CV) disease, particularly coronary artery disease (CAD), heart failure (HF) and peripheral arterial disease. Over the past decades, the incidence of diabetes has greatly increased. Type 2 diabetes is a common condition, affecting an estimated 537 million people worldwide in 2021 (10.5% prevalence), a number expected to rise to 783 million by 2045 (12.2% prevalence).^1^ The growing prevalence, especially among younger individuals, is particularly concerning and is related to increasing rates of overweight and obesity. Moreover, longer duration of diabetes, and the resulting prolonged exposure to hyperglycaemia, is considered a key factor contributing to the development of diabetes-related complications such as CV disease.^2^

In parallel, the proportion of patients presenting with both myocardial infarction (MI) and diabetes is increasing, with a prevalence of 20%–30%.^3^ Type 2 diabetes among patients with MI is associated with a higher risk of mortality and recurrent ischaemic events. Targeting hyperglycaemia alone does not appear to reduce the risk of repeat macrovascular events.^4 5^ However, sodium-glucose transport protein 2 (SGLT2) inhibitors and glucagon-like peptide-1 receptor agonists (GLP-1 RA) with several modes of action have been shown to reduce the CV risk in patients with type 2 diabetes and established CV disease, including MI.^6 7^

A substantial proportion of patients with MI without known diabetes have disturbed glucose metabolism, including pre-diabetes, manifesting as impaired fasting glucose (IFG) or impaired glucose tolerance (IGT).^8^ For long there has been evidence that the risk for atherosclerotic events/macrovascular disease starts even below the level of the diabetes cut-off value.^9^ However, the impact of new-onset diabetes and intermediate glucose disturbances after MI on long-term CV outcomes and mortality remains unclear. The clinical significance of detecting these abnormalities in the subacute phase after MI, their incremental risk beyond traditional risk factors, and whether they warrant early targeted intervention remain uncertain.

In this nationwide, real-world cohort of patients with MI, the aim was to examine the prevalence of pre-diabetes and new-onset diabetes, assessed at the 2-month post-MI cardiac rehabilitation visit, and to evaluate their associations with CV outcomes and mortality.

## Methods

### Study population

**Figure 1:**
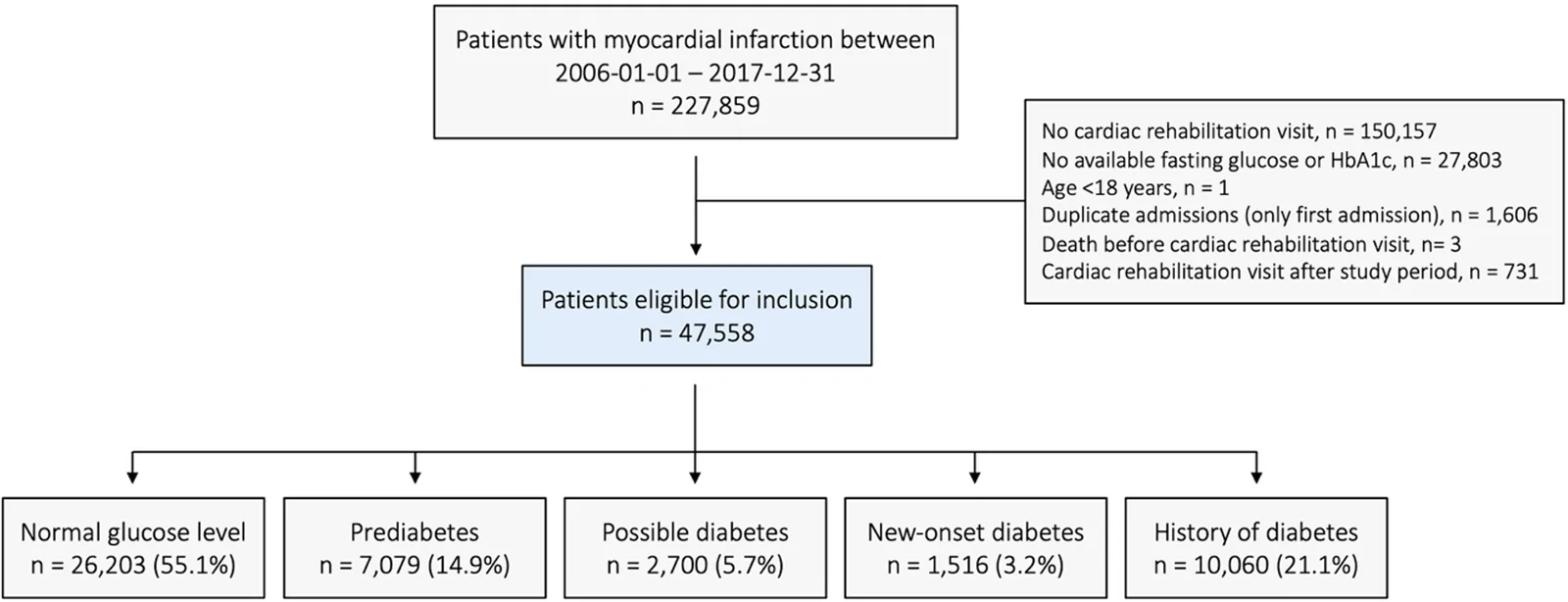
Patient inclusion flowchart. HbA1c, glycated haemoglobin.

This is a retrospective observational study including patients with a first cardiac rehabilitation follow-up visit after a first MI between 1 January 2006 and 31 December 2017, using data from the Swedish Web-system for Enhancement and Development of Evidence-based care in Heart disease Evaluated According to Recommended Therapies (SWEDEHEART) registry. All patients admitted with an MI to any of the 74 cardiac care units in Sweden are registered in the national quality registry. All patients aged over 18 and under 75 years who were eligible for and attended a first follow-up visit within the cardiac rehabilitation programme approximately 2 months (6–10 weeks) after discharge were included (figure 1). Laboratory data, including fasting plasma glucose for all patients and, in many cases, glycated haemoglobin (HbA1c), were analysed at the local hospital laboratories according to standardised methods. The SWEDEHEART registry was cross-referenced with mandatory national health registers, including the National Patient Register, which captures hospitalisation diagnoses, the National Cause of Death Register and the National Prescribed Drug Register, containing data on all dispensed prescription drugs. The completeness and accuracy of SWEDEHEART have previously been reported with continuous monitoring to ensure high validity.^10^ All variables and definitions are reported in online supplemental table 1.

### Exposure variables

Blood samples were drawn in a fasting state 1–2 weeks prior to the cardiac rehabilitation visit. Based on the fasting glucose level, HbA1c and history of diabetes, the following exposure groups were defined: (1) no diabetes with normal plasma glucose and HbA1c according to WHO diabetes criteria^11^; (2) no history of diabetes but with pre-diabetes (plasma glucose 6.1–6.9 mmol/L and/or HbA1c 42–47 mmol/mol); (3) no clinical diagnosis of diabetes but hyperglycaemia indicating possible diabetes (plasma glucose ≥7.0 mmol/L and/or HbA1c ≥48 mmol/mol); (4) new-onset diabetes, defined as diabetes diagnosed during MI hospitalisation or between hospital discharge and index visit; and (5) an established diabetes diagnosis prior to MI hospitalisation.

### Outcomes

The primary outcome was major adverse CV events (MACE), a composite of all-cause mortality, MI and ischaemic stroke. Secondary outcomes included all the components of MACE, CV mortality and hospitalisation for heart failure. Detailed outcome definitions are available in online supplemental table 1. The study index date was the 2-month cardiac rehabilitation visit date (6–10 weeks after discharge for MI), and complete follow-up was available until a fatal event occurred or 30 June 2018.

### Statistical analyses

The findings were presented following the Strengthening the Reporting of Observational Studies in Epidemiology (STROBE) guidelines. Continuous variables are presented as medians with IQRs. Categorical variables are presented as counts and percentages. Demographics and other characteristics were compared across exposure strata. Kaplan-Meier survival plots were used to present outcomes in relation to the exposure, with the corresponding number at risk provided. Fasting plasma glucose and HbA1c were analysed both as categorical variables using predefined WHO-based glycaemic categories and as continuous variables using restricted cubic splines. Age and body mass index (BMI) were included as continuous variables in the regression models.

Unadjusted and adjusted Cox proportional hazards models were fitted for the entire follow-up and were presented using glycaemic status as a categorical variable. Two models were fitted: (1) unadjusted and (2) adjusted for age, sex, BMI, smoking, hypertension, hyperlipidaemia, physical activity, history of stroke, prior MI, prior percutaneous coronary intervention (PCI), prior coronary artery bypass grafting (CABG) and heart failure. Relationships between exposure and outcomes were depicted in forest plots. In a sensitivity analysis, chronic kidney disease (CKD) was added as an additional covariate to the adjusted model. A further sensitivity analysis was performed in which patients classified as possible diabetes and new-onset diabetes were combined into a single category. Multiple imputation of missing values for patient characteristics and comorbidities was performed using an arbitrary missing pattern. We assumed that missingness is conditional on age, sex, BMI, smoking, hypertension, hyperlipidaemia, physical activity, history of stroke, prior MI, prior PCI, prior CABG and heart failure. The SAS function PROC MI was used, and five imputed datasets were generated to ensure that our effect estimates would not overlay inaccurately due to Monte Carlo variability.^12^ A fully conditional specification (FCS) statement was used, which specifies a multivariate imputation by fully conditional specification methods. To impute categorical variables with the FCS method, logistic regression models were used for binary variables and Predictive Mean Matching (regpredmeanmatch) for continuous variables. The results for each imputation were combined using the SAS function PROC MIANALYZE. In addition, the relationship between levels of fasting plasma glucose and HbA1c at the time point or cardiac rehabilitation visit was presented using multivariate regression with restricted cubic splines to allow for non-linearity. A two-sided p-value of <0.05 was considered statistically significant. All analyses were performed at the Uppsala Clinical Research Centre (UCR), Uppsala University, Uppsala, Sweden, using SAS Software (SAS Institute, Cary, North Carolina, USA).

### Patient and public involvement

None.

## Results

### Baseline characteristics

In total, 47 558 patients were included and stratified by glycaemic category (figure 1). pre-diabetes (14.9%) and possible diabetes (5.7%) were common and occurred at rates almost comparable to established diabetes (21.1%). The median age was 64 years and increased progressively across categories, from 63 years in individuals with no diabetes to 66 years in those with diabetes diagnosed prior to the index MI hospitalisation (table 1). Women comprised 26% of the total population, with a higher proportion among those with new-onset diabetes or a history of diabetes. A stepwise increase in BMI was observed with worsening glycaemic status, from 26 kg/m² in those with no diabetes to 29 kg/m² in patients with a history of diabetes. The burden of comorbidities increased progressively across glycaemic categories (table 1). The use of glucose-lowering treatment reflected the accompanying category. In those with a history of diabetes, 41.7% received insulin, 54.2% received metformin, and smaller proportions were treated with SGLT2 inhibitors (1.3%), GLP-1 RA (2.3%) or dipeptidyl peptidase-4 inhibitors (5.0%).

**Table 1:** Baseline demographic and clinical characteristics

Characteristics	Normal glucose level (n=26 203)	Pre-diabetes (n=7079)	Possible diabetes (n=2700)	New-onset diabetes (n=1516)	History of diabetes (n=10 060)	All patients (n=47 558)
Demographics						
Age, years, median (IQR)	63 (56–69)	64 (56–69)	65 (58–69)	66 (60–70)	66 (59–70)	64 (57–69)
Sex, women, n (%)	6674 (25.5)	1587 (22.4)	609 (22.6)	516 (34)	2833 (28.2)	12 219 (25.7)
Smoker, n (%)	2807 (10.7)	800 (11.3)	325 (12.1)	315 (20.8)	321 (13.2)	5568 (11.7)
Body mass index, kg/m^2^, median (IQR)	26 (13–154)	28 (16–105)	28 (15–109)	27 (14–54)	29 (12–261)	27 (12–261)
Medical history, n (%)						
Hypertension	12 104 (46.2)	3706 (52.4)	1604 (59.4)	835 (55.1)	7874 (78.3)	26 123 (54.9)
Prior myocardial infarction	2936 (11.2)	882 (12.5)	411 (15.2)	198 (13.1)	2841 (28.2)	7268 (15.3)
Prior ischaemic stroke	634 (2.4)	182 (2.6)	85 (3.1)	47 (3.1)	648 (6.4)	1596 (3.4)
Heart failure	2714 (10.4)	883 (12.5)	418 (15.5)	282 (18.6)	2288 (22.7)	6585 (13.8)
Peripheral vascular disease	4735 (18.1)	1352 (19.1)	581 (21.5)	336 (22.2)	3395 (33.7)	10 399 (21.9)
Chronic kidney disease	430 (1.6)	107 (1.5)	72 (2.7)	30 (2.0)	737 (7.3)	1376 (2.9)
Chronic obstructive pulmonary disease	751 (3)	182 (3.0)	87 (3.0)	897 (59.0)	1622 (16.0)	3539 (7.0)
Cancer (within 3 years)	857 (3.3)	293 (4.1)	139 (5.1)	62 (4.1)	638 (6.3)	1989 (4.2)
Dementia	34 (0.1)	8 (0.1)	6 (0.2)	5 (0.3)	18 (0.2)	71 (0.1)
Index event and biomarker levels, n (%)						
ST-segment elevation myocardial infarction	10 948 (41.8)	3034 (42.9)	1079 (40.0)	591 (39.0)	3239 (32.2)	18 891 (39.7)
Coronary angiography	25 548 (97.5)	6946 (98.1)	2612 (96.7)	1465 (96.6)	9462 (94.1)	46 033 (96.8)
Percutaneous coronary intervention	21 547 (82.2)	6062 (85.6)	2254 (83.5)	1174 (77.4)	7604 (75.6)	38 641 (81.3)
Coronary artery bypass grafting	1141 (4.4)	367 (5.2)	155 (5.7)	64 (4.2)	1171 (11.6)	2898 (6.1)
Fasting plasma-glucose, mmol/L, median (IQR)	5.4 (5.1–5.7)	6.3 (6.2–6.5)	7.4 (7.1–7.9)	6.0 (5.4–7.1)	7.7 (6.3–9.6)	5.8 (5.3–6.6)
Glycated haemoglobin, mmol/mol, median (IQR)	37 (35–40)	44 (43–45)	46 (42–50)	46 (40–53)	55 (48–66)	43.00 (38 – 55)
Medication, n (%)						
Insulin	0 (0.0)	0 (0.0)	0 (0.0)	21 (1.4)	4191 (41.7)	4212 (8.9)
Metformin	0 (0.0)	0 (0.0)	0 (0.0)	223 (14.7)	5450 (54.2)	5673 (11.9)
Sodium-glucose transport protein 2 inhibitors	0 (0.0)	0 (0.0)	0 (0.0)	2 (0.1)	133 (1.3)	135 (0.3)
Glucagon-like peptide-1 receptor agonists	0 (0.0)	0 (0.0)	0 (0.0)	0 (0.0)	235 (2.3)	235 (0.5)
Dipeptidyl peptidase-4 inhibitors	0 (0.0)	0 (0.0)	0 (0.0)	5 (0.3)	500 (5.0)	505 (1.1)
Sulfonylureas	0 (0.0)	0 (0.0)	0 (0.0)	7 (0.5)	1382 (13.7)	1389 (2.9)
Lipid-lowering therapy	25 882 (98.8)	7030 (99.3)	2672 (99.0)	1497 (98.7)	10 002 (99.4)	47 083 (99.0)

Values are in median (IQR) for continuous variables and numbers (%) for categorical variables.

### Glycaemic categories and MACE

**Figure 2:**
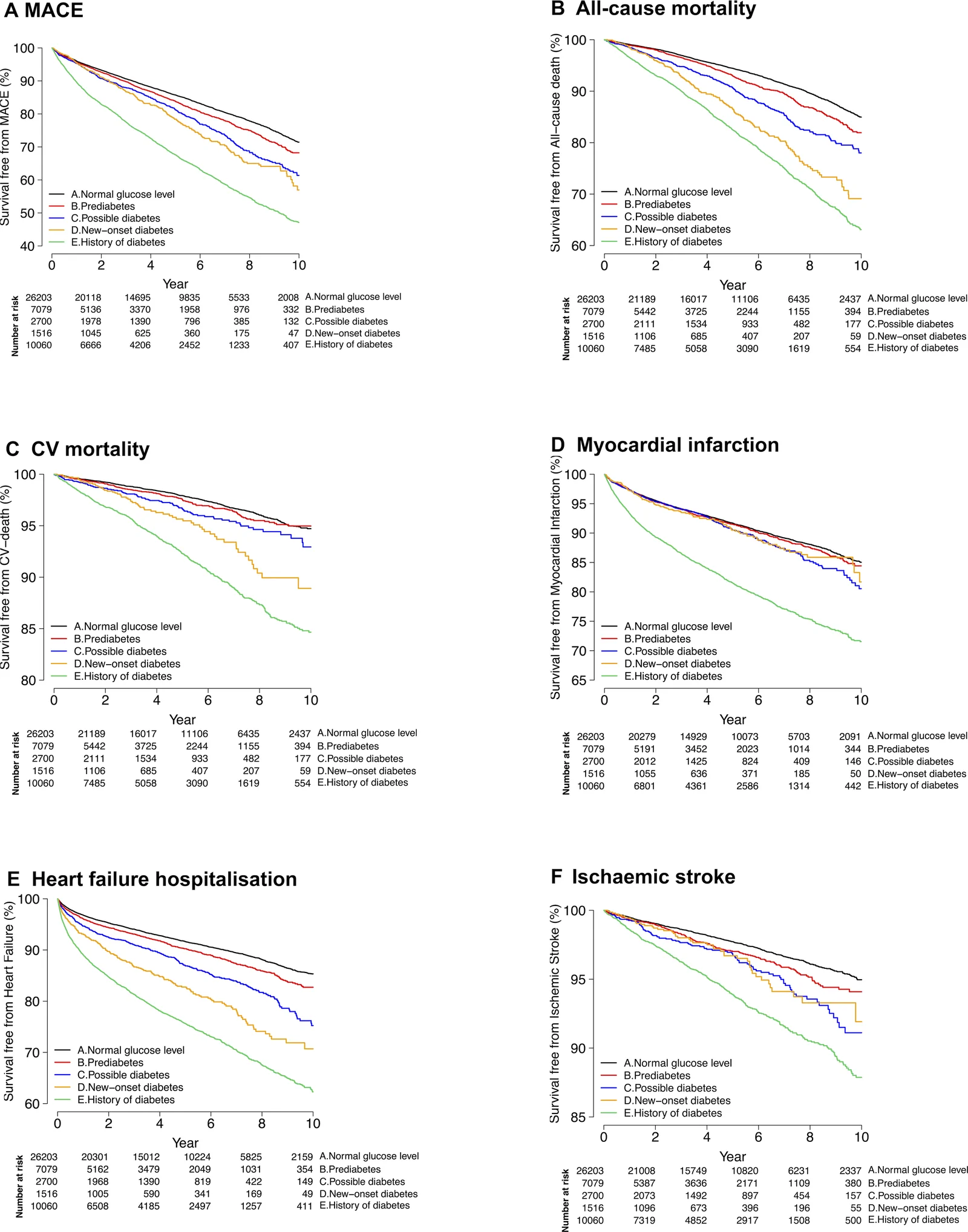
Kaplan-Meier estimates and long-term risk of MACE, mortality and CV outcomes in relation to glycaemic status at index visit post-myocardial infarction. CV, cardiovascular; MACE, major adverse CV event.

**Figure 3:**
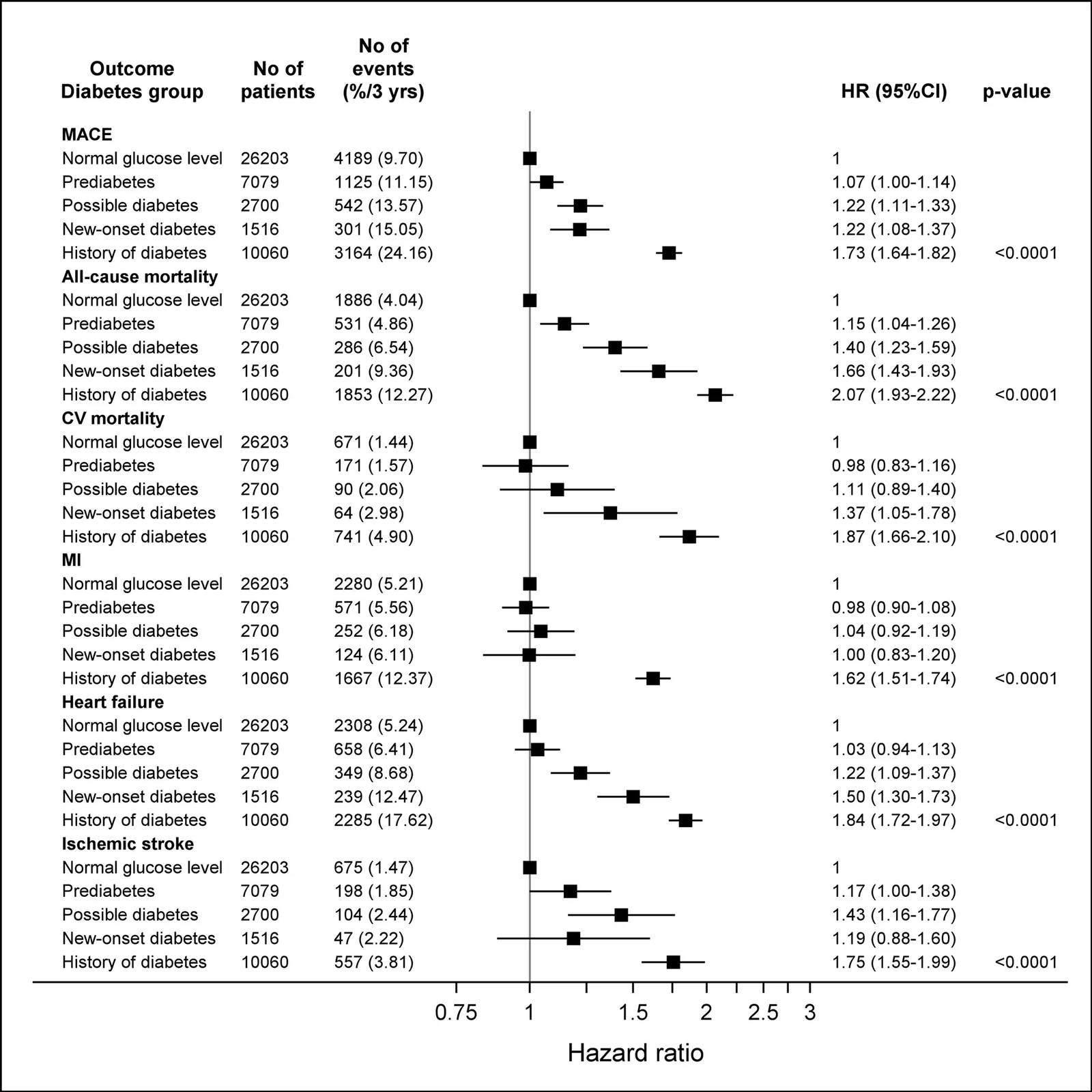
Long-term risk of MACE, mortality and CV outcomes in relation to glycaemic category at index visit post-MI. Forest plot with adjusted Cox regression HRs (95% CIs) with no diabetes and with normal glucose level as reference. CV, cardiovascular; MACE, major adverse CV event; MI, myocardial infarction.

The median follow-up time from the study index to the end of follow-up was 4.7 years, during which 9321 patients experienced MACE. Kaplan-Meier estimates by each category are presented in figure 2. Rates of MACE increased progressively in different glycaemic categories with early curve separation. There was a stepwise increase in MACE risk across exposure strata in both unadjusted (online supplemental figure 1) and adjusted analyses (figure 3). For example, pre-diabetes versus normal glucose levels (HR 1.07, 95% CI 1.00 to 1.14), possible diabetes versus normal glucose levels (HR 1.22, 95% CI 1.11 to 1.33), new-onset diabetes versus normal glucose levels (HR 1.22, 95% CI 1.08 to 1.37) and history of diabetes versus normal glucose levels (HR 1.73, 95% CI 1.64 to 1.82). In a sensitivity analysis, adding CKD as a covariate had minimal impact on the observed findings (online supplemental table 2). Furthermore, when possible diabetes and new-onset diabetes were combined into a single category, the results remained unchanged. This combined group had an increased risk of MACE (HR 1.22, 95% CI 1.13 to 1.31), all-cause mortality (HR 1.49, 95% CI 1.35 to 1.66), heart failure hospitalisation (HR 1.32, 95% CI 1.20 to 1.45), CV mortality (HR 1.21, 95% CI 1.01 to 1.45) and ischaemic stroke (HR 1.35, 95% CI 1.12 to 1.61).

### Glycaemic categories and secondary events

Kaplan-Meier estimates for all secondary outcomes, including all-cause mortality, CV mortality, MI, heart failure hospitalisation and ischaemic stroke, are presented in figure 2. Similar to MACE, stepwise worsening glycaemic category was associated with an increased rate of all secondary outcomes, except for recurrent MI. In the adjusted model, possible diabetes, new-onset diabetes and a history of diabetes were each associated with an increased risk of all-cause mortality, CV mortality, heart failure hospitalisation and ischaemic stroke (figure 3). In contrast, an increased risk of MI was observed only among patients with a history of diabetes compared with those with no diabetes.

### Plasma glucose and HbA1c levels and risk of events

**Figure 4:**
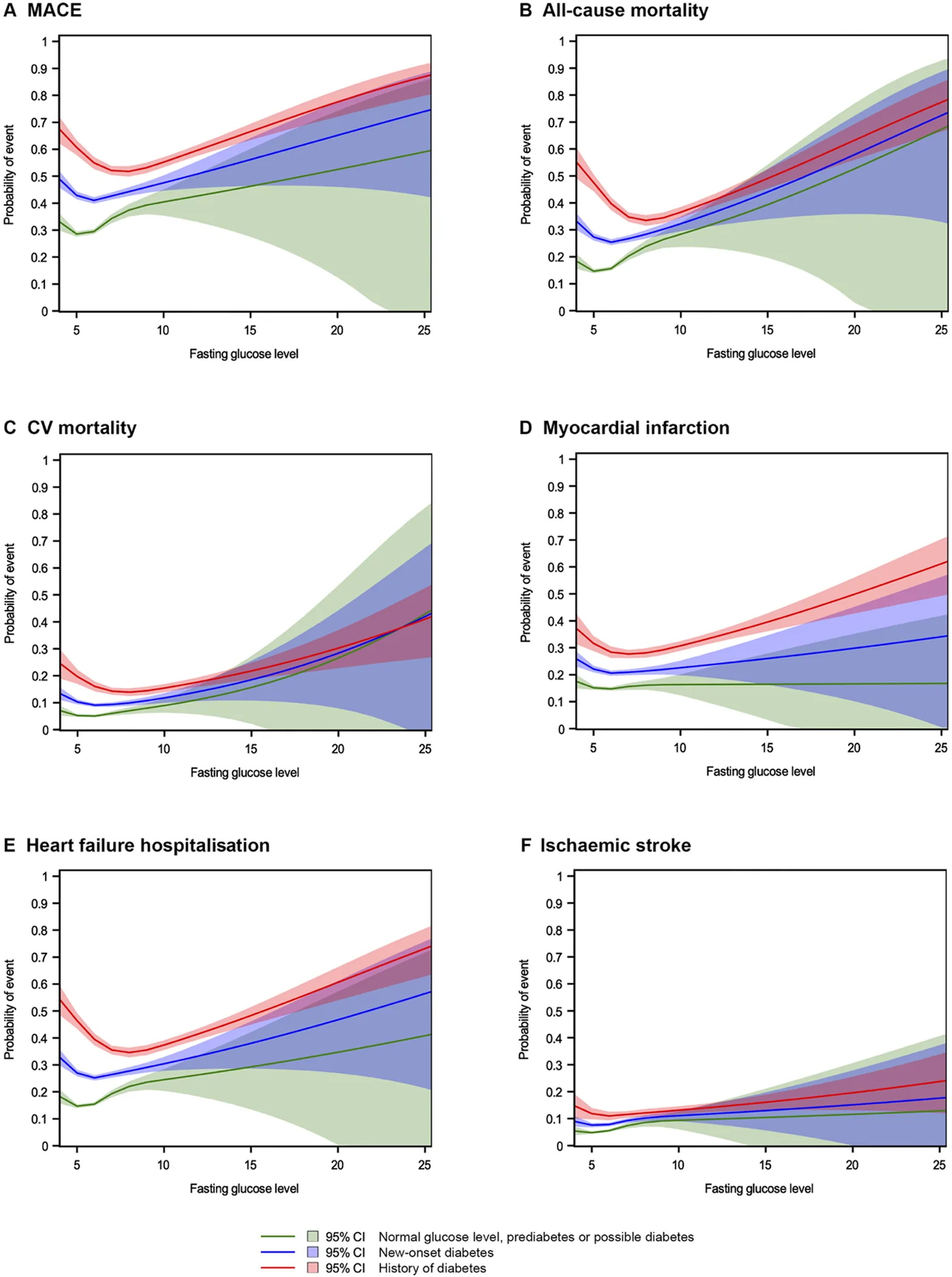
Fasting glucose (mmol/L) at index visit post-myocardial infarction and long-term risk of MACE, mortality and CV outcomes. CV, cardiovascular; MACE, major adverse CV event.

Using restricted cubic splines, a near-linear association was observed between increasing fasting plasma glucose levels from 7 mmol/L upwards at study index and the risk of MACE, all-cause mortality, CV mortality and heart failure hospitalisation, irrespective of diabetes status (figure 4). Among patients without diabetes, the increase in risk began below the glucose threshold for diabetes. For MI, the increased risk associated with higher fasting glucose levels was most pronounced among patients with a history of diabetes. HbA1c data were available for 13,994 (30%) of the population. In contrast, the association between higher levels of HbA1c and increased risk of MACE was mainly observed in patients with a history of diabetes (online supplemental figure 2).

## Discussion

In this nationwide cohort study, we examined the association between early postdischarge glycaemic status following MI, including fasting glucose and HbA1c levels, and adverse outcomes. A stepwise increase was observed, with known diabetes exhibiting the highest risk for all major CV outcomes, followed by newly diagnosed diabetes, possible diabetes and pre-diabetes. Notably, patients classified as having possible diabetes exhibited a risk similar to that of new-onset diabetes, suggesting that individuals fulfilling biochemical criteria for diabetes may already carry a CV risk comparable to those with clinically recognised diabetes. A near-linear association was observed between fasting glucose and outcomes across all patients, while HbA1c was associated with adverse outcomes primarily in those with established diabetes. This difference may reflect the different biological processes captured by these biomarkers. Hyperglycaemia measured following MI reflects current metabolic and neurohormonal disturbances, whereas HbA1c reflects cumulative glycaemic exposure and the long-term vascular consequences of chronic hyperglycaemia, which may be particularly relevant in established diabetes.^13 14–17^ Nevertheless, these findings underscore the prognostic significance of both glucose and HbA1c levels after MI, even when values are within the normal range.

Prior studies have shown that patients with diabetes experience worse outcomes after MI, including higher rates of recurrent ischaemic events, heart failure and mortality.^18^ A large individual participant meta-analysis demonstrated an association between fasting glucose concentrations and coronary heart disease in individuals without established vascular disease, with a near-linear relationship.^9^ Our study extends these observations to a post-MI population by distinguishing between various glycaemic states, such as pre-diabetes, possible diabetes and new-onset diabetes, and by demonstrating that fasting glucose and HbA1c provide complementary rather than interchangeable prognostic information following MI. Our findings highlight the importance of early identification of dysglycaemia following MI to improve risk stratification. Whether targeted interventions in these populations improve outcomes requires prospective evaluation.

SGLT2 inhibitors and GLP-1 RAs have proven CV benefit in patients with diabetes and established heart disease, such as MI.^6 7^ However, the use of these medications was uncommon in our cohort, as it predates the publication of guidelines following some of the CV outcome trials that highlighted their benefits.^19 20^ Consequently, we are currently unable to assess the potential benefits and risks associated with these drugs within our study population. Nevertheless, the increased CV risk observed among patients with pre-diabetes and possible diabetes raises the question of whether these groups might benefit from therapies currently indicated for diabetes. This hypothesis requires evaluation in prospective studies.

The findings in our study have several clinical implications. First, our results highlight the importance of routine assessment of fasting glucose and HbA1c in patients post-MI, since previously unrecognised diabetes can be identified, and the detection of pre-diabetes also appears important, given the high risk of CV events in these patients. Current clinical practice guidelines recommend aggressive risk factor management in patients with diabetes; however, patients with pre-diabetes or possible diabetes may also benefit from closer monitoring and CV risk assessment.^21^ In addition, CV risk following MI is influenced by multiple modifiable factors, including lipid levels and the use of guideline-directed secondary prevention therapies. Although our study focused on glycaemic status, the interpretation of metabolic risk profiles should consider the contribution of comprehensive risk-factor management, including lipid-lowering strategies.^22^ Lifestyle modifications, pharmacological interventions and comprehensive CV risk management remain fundamental components of secondary prevention, especially in patients with newly detected diabetes. Second, the risk of heart failure hospitalisation was elevated among patients with pre-diabetes, possible diabetes and those with a clinical diabetes diagnosis, warranting closer surveillance.^18 23–26^

A strength of this study was the use of the SWEDEHEART registry, which provides comprehensive, high-quality national data on patients with MI. The large sample size, data from the cardiac rehabilitation visit and the long follow-up period enhanced the reliability of our findings. Additionally, our study included a well-defined classification of glycaemic categories, allowing for a nuanced assessment of diabetes status and its impact on CV outcomes. However, some limitations must be acknowledged. First, as a retrospective observational study, causality cannot be established. While we adjusted for multiple confounders, residual confounding remains possible. Second, fasting glucose was only measured at one time point, which may not fully capture glycaemic variability over time. The timing of the first cardiac rehabilitation visit varied between 6 and 10 weeks after MI, which may have introduced some variability in metabolic measurements due to differences in recovery phase, lifestyle changes and early post-MI treatment optimisation. However, any resulting misclassification of glycaemic categories would most likely have been non-differential and therefore expected to attenuate, rather than exaggerate, the observed associations. Third, as the study population was limited to patients attending the cardiac rehabilitation programme, patients who did not attend could not be evaluated. Consequently, selection and survivor bias cannot be excluded, and the findings may not be fully generalisable to the broader post-MI population. Fourth, HbA1c was not a mandatory variable, potentially leading to missing data and selection bias. Fifth, the category ‘possible diabetes’ fulfils epidemiological criteria for diabetes but was not included in the ‘new-onset diabetes’ group, as no clinical diagnosis was established. This may have led to misclassification and an underestimation of the burden of new-onset diabetes in the cohort. This also limits the ability to directly compare our findings with studies applying the epidemiological definitions of diabetes, potentially affecting the interpretation of risk associated with early-stage or undiagnosed disease. As oral glucose tolerance test (OGTT) data were not available in our study, fasting glucose and HbA1c were used to classify the exposure variable. However, if OGTT had been performed routinely, it might have identified additional cases of previously unrecognised diabetes that would not have been detected by fasting plasma glucose or HbA1c alone.^27^ Furthermore, we did not assess the impact of specific antidiabetic treatments on CV outcomes, which could influence the observed associations.

## Conclusions

This study demonstrates a graded association between increasing glycaemic disturbance and adverse CV outcomes following an MI. Patients with pre-diabetes, possible diabetes or new-onset diabetes are at elevated risk, underscoring the importance of early detection, comprehensive glucose monitoring and intensified CV risk management. Future studies should assess whether targeted glucose-lowering strategies can improve long-term outcomes, particularly in early-stage disease.

## Supplementary material

10.1136/bmjopen-2026-121970 online supplemental file 1

## Data Availability

Data are available upon reasonable request.
