# Supplementary material for: Diabetes status, fasting glucose and HbA1c, and outcomes after myocardial infarction: a retrospective registry-based Swedish cohort study
Source: BMJ Open. 2026 Sep 18;16(9):e121970. doi: 10.1136/bmjopen-2026-121970 (PMC13599995; doi:10.1136/bmjopen-2026-121970)

Supplementary material

Diabetes status, fasting glucose and HbA1c, and outcomes after myocardial infarction: a retrospective registry-based Swedish cohort study

Supplementary Table 1. Data sources and the International Code of Disease, tenth revision (ICD-10) and the Anatomical Therapeutic Chemical (ATC) codes applied to identify comorbidities and comedication.

Variable	Data source	ICD-10 / ATC code
Age	SWEDEHEART	-
Sex	SWEDEHEART	-
Smoker	SWEDEHEART	-
BMI	SWEDEHEART	-
Hypertension	SWEDEHEART, NPR	I10, I11, I12, I13, I15
Diabetes mellitus	SWEDEHEART, NPR	E10, E11, E12, E13, E14
Prior MI	SWEDEHEART, NPR	I21, I22, I252
Prior stroke	SWEDEHEART, NPR	I60, I61, I63, I64
Prior systemic embolism	NPR	I74
Heart failure	SWEDEHEART, NPR	I42, I50, I110, I255, I130, I132, K761
Peripheral arterial disease	NPR	I70, I71, I72, I73
Chronic kidney disease	NPR	N17, N18
COPD	NPR	J43, J44
Cancer (within 3 years)	NPR	C
Dementia	NPR	F00, F01, F02, F03, F051, G30, G311, G318A
Type of MI (NSTEMI, STEMI)	SWEDEHEART	-
HbA1c	SWEDEHEART	-
Creatinine	SWEDEHEART	-
Coronary angiography	SWEDEHEART	-
PCI	SWEDEHEART	-
CABG	SWEDEHEART	-
Insulin	SWEDEHEART, NPDR	A10A
Metformin	SWEDEHEART, NPDR	A10BA02
SGLT2 inhibitors	SWEDEHEART, NPDR	A10BK
GLP-1 receptor agonists	SWEDEHEART, NPDR	A10BJ
DPP-4 inhibitors	SWEDEHEART, NPDR	A10BH
Sulfonylureas	SWEDEHEART, NPDR	A10BB

Abbreviations: BMI, body mass index; ATC, anatomical therapeutic chemical; CABG, coronary artery bypass grafting; COPD, chronic obstructive pulmonary disease; DPP-4, dipeptidyl peptidase-4; GLP-1, glucagon-like peptide-1; IQR, interquartile range; MI, myocardial infarction; NPDR, national prescribed drug register; NPR, national patient register; NSTEMI, non-ST- ST-segment elevation myocardial infarction; SGLT2, sodium-glucose transport protein 2; STEMI, ST-segment elevation myocardial infarction.

Supplementary Figure 1. Long-term risk of MACE, mortality and CV outcomes in relation to glycaemic category at index visit post-MI. Forest plot with unadjusted Cox regression hazard ratios (95% CIs) with no diabetes with normal glucose level as reference.

Outcome<br>Diabetes group	No of patients	No of events (%/3 yrs)	HR (95%CI)	p-value
**MACE**				
Normal glucose level	26203	4189 (9.70)	1 (Ref.)	
Prediabetes	7079	1125 (11.15)	1.15 (1.07-1.23)	
Possible diabetes	2700	542 (13.57)	1.40 (1.28-1.53)	
New-onset diabetes	1516	301 (15.05)	1.54 (1.37-1.73)	
History of diabetes	10060	3164 (24.16)	2.47 (2.36-2.59)	<0.0001
**All-cause mortality**				
Normal glucose level	26203	1886 (4.04)	1 (Ref.)	
Prediabetes	7079	531 (4.86)	1.25 (1.13-1.37)	
Possible diabetes	2700	286 (6.54)	1.67 (1.47-1.89)	
New-onset diabetes	1516	201 (9.36)	2.45 (2.12-2.83)	
History of diabetes	10060	1853 (12.27)	3.15 (2.96-3.36)	<0.0001
**CV mortality**				
Normal glucose level	26203	671 (1.44)	1 (Ref.)	
Prediabetes	7079	171 (1.57)	1.11 (0.93-1.31)	
Possible diabetes	2700	90 (2.06)	1.45 (1.16-1.81)	
New-onset diabetes	1516	64 (2.98)	2.12 (1.64-2.74)	
History of diabetes	10060	741 (4.90)	3.46 (3.12-3.84)	<0.0001
**MI**				
Normal glucose level	26203	2280 (5.21)	1 (Ref.)	
Prediabetes	7079	571 (5.56)	1.04 (0.95-1.14)	
Possible diabetes	2700	252 (6.18)	1.16 (1.02-1.33)	
New-onset diabetes	1516	124 (6.11)	1.12 (0.94-1.35)	
History of diabetes	10060	1667 (12.37)	2.29 (2.15-2.44)	<0.0001
**Heart failure**				
Normal glucose level	26203	2308 (5.24)	1 (Ref.)	
Prediabetes	7079	658 (6.41)	1.18 (1.08-1.29)	
Possible diabetes	2700	349 (8.68)	1.61 (1.44-1.81)	
New-onset diabetes	1516	239 (12.47)	2.22 (1.95-2.54)	
History of diabetes	10060	2285 (17.62)	3.18 (3.00-3.37)	<0.0001
**Ischaemic stroke**				
Normal glucose level	26203	675 (1.47)	1 (Ref.)	
Prediabetes	7079	198 (1.85)	1.27 (1.08-1.48)	
Possible diabetes	2700	104 (2.44)	1.67 (1.36-2.05)	
New-onset diabetes	1516	47 (2.22)	1.53 (1.14-2.05)	
History of diabetes	10060	557 (3.81)	2.61 (2.34-2.92)	<0.0001

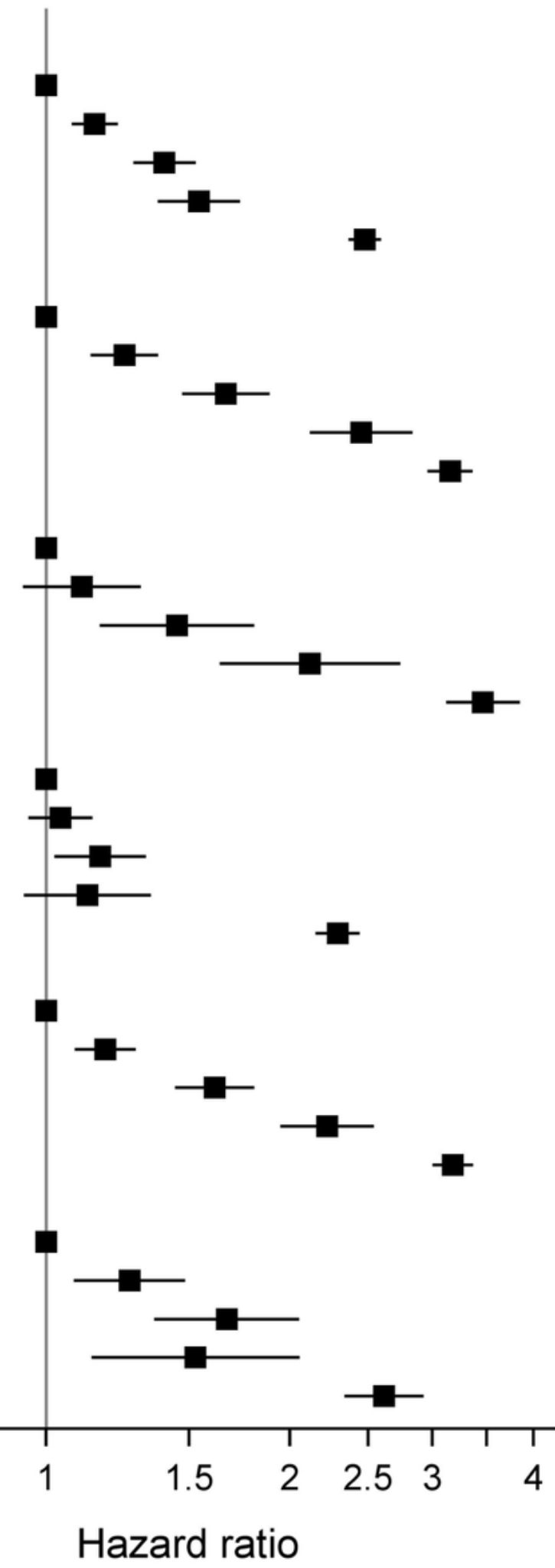

Supplementary Table 2. Long term risk of MACE, mortality and CV outcomes in relation to glycaemic category at index visit post-MI. Fully adjusted Cox proportional hazards ratios (95%CIs) with no diabetes and with normal glucose as reference, with additional adjustment for chronic kidney disease

Outcome	Diabetes groups	Number of patients	Events (%/3 yr)	HR (95% CI)	p-value
**MACE**	Normal glucose level	26203	4189 (9.70)	1	
	Prediabetes	7079	1125 (11.15)	1.07 (1.01-1.15)	
	Possible diabetes	2700	542 (13.57)	1.22 (1.12-1.34)	
	New-onset diabetes	1516	301 (15.05)	1.23 (1.10-1.38)	
	History of diabetes	10060	3164 (24.16)	1.70 (1.62-1.79)	<.0001
**All-cause mortality**	Normal glucose level	26203	1886 (4.04)	1	
	Prediabetes	7079	531 (4.86)	1.16 (1.05-1.27)	
	Possible diabetes	2700	286 (6.54)	1.40 (1.24-1.59)	
	New-onset diabetes	1516	201 (9.36)	1.69 (1.46-1.97)	
	History of diabetes	10060	1853 (12.27)	2.02 (1.88-2.17)	<.0001
**CV mortality**	Normal glucose level	26203	671 (1.44)	1	
	Prediabetes	7079	171 (1.57)	0.99 (0.84-1.17)	
	Possible diabetes	2700	90 (2.06)	1.12 (0.90-1.41)	
	New-onset diabetes	1516	64 (2.98)	1.40 (1.08-1.82)	
	History of diabetes	10060	741 (4.90)	1.83 (1.63-2.06)	<.0001
**MI**	Normal glucose level	26203	2280 (5.21)	1	
	Prediabetes	7079	571 (5.56)	0.99 (0.90-1.08)	
	Possible diabetes	2700	252 (6.18)	1.05 (0.92-1.20)	
	New-onset diabetes	1516	124 (6.11)	1.00 (0.84-1.21)	
	History of diabetes	10060	1667 (12.37)	1.56 (1.49-1.71)	<.0001
**Heart failure**	Normal glucose level	26203	2308 (5.24)	1	
	Prediabetes	7079	658 (6.41)	1.034 (0.95-1.13)	
	Possible diabetes	2700	349 (8.68)	1.23 (1.09-1.38)	
	New-onset diabetes	1516	239 (12.47)	1.51 (1.31-1.74)	
	History of diabetes	10060	2285 (17.62)	1.82 (1.70-1.94)	<.0001
**Ischaemic stroke**	Normal glucose level	26203	675 (1.47)	1	
	Prediabetes	7079	198 (1.85)	1.16 (1.00-1.38)	
	Possible diabetes	2700	104 (2.44)	1.43 (1.16-1.77)	
	New-onset diabetes	1516	47 (2.22)	1.20 (0.89-1.61)	
	History of diabetes	10060	557 (3.81)	1.74 (1.54-1.97)	<.0001

Supplementary Figure 2. HbA1c (mmol/mol) at index visit post-MI and long-term risk of MACE, mortality and CV outcomes. A total of 13,994 patients with available HbA1c data were included in this analysis.

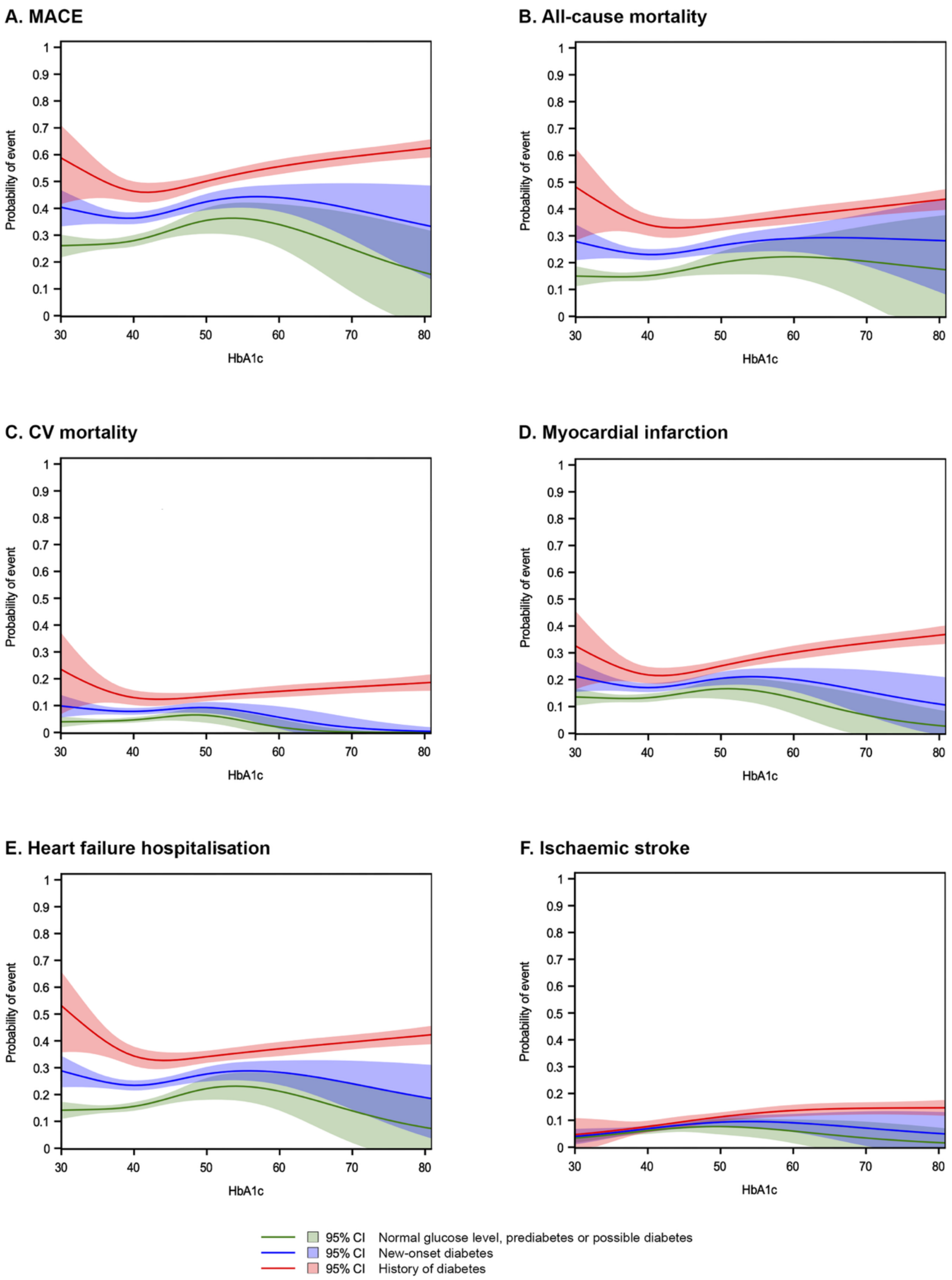

Supplement: Supplementary file 1 [file bmjopen-16-9-s001.pdf]
