# Supplementary material for: Diabetes status, fasting glucose and HbA1c, and outcomes after myocardial infarction: a retrospective registry-based Swedish cohort study
Source: BMJ Open. 2026 Sep 18;16(9):e121970. doi: 10.1136/bmjopen-2026-121970 (PMC13599995; doi:10.1136/bmjopen-2026-121970)
Supplement: Supplementary file 2 [file bmjopen-2026-121970.reviewer_comments.pdf]

PEER REVIEW HISTORY

BMJ Open publishes all reviews undertaken for accepted manuscripts. Reviewers are asked to complete a checklist review form and are provided with free text boxes to elaborate on their assessment. These free text comments are reproduced below.

ARTICLE DETAILS

Title (Provisional)

Diabetes status, fasting glucose and HbA1c, and outcomes after myocardial infarction: a retrospective registry-based Swedish cohort study

Authors

Haider, Azzum; Hagström, Emil; Ishak, Divan; Hadziosmanovic, Nermin; Norhammar, Anna; Velders, Matthijs; Batra, Gorav

---

VERSION 1 - REVIEW

**Reviewer**	**1**
**Name**	**De Backer, Guy**
**Affiliation**	**University of Ghent**
**Date**	**04-Jun-2026**
**COI**	**None**

In this manuscript entitled " Diabetes status, fasting glucose and HbA1c, and outcomes after myocardial infarction: nationwide cohort study" the prevalences of pre-diabetes and of new-onset diabetes type 2 were examined in post-MI patients who attended a cardiac rehabilitation (CR programme in Sweden between 2006 and 2017. Associations between dysglycaemia and CV outcomes and mortality are also reported. The results are well presented and the manuscript reads well.

Comments:

- The study is announced as a ' nationwide cohort study'. One of the strengths is indeed the use of a high-quality national data base to start with. However only patients who attended a CR programme were included (47558 out of 227859). This selection may well have influenced the results. It is well known that referral to and attendance of CR programs by patients with CHD is limited in many countries. From presentations at international meetings I recall that in the EUROASPIRE V survey only 74% of eligible patients with CHD were advised to attend a CR programme in Sweden and only 65% of those advised did attend CR. Patients wo attend CR are in general better off than non-participants. Therefore the prevalences of pre-diabetes and of new-onset diabetes in this study may well underestimate the real-life prevalences in coronary patients.

- Dysglycaemia was examined in this study using fasting glucose levels (FGL) and HbA1c levels at baseline. It has been shown that in patients with CHD the use of an oral glucose tolerance test (OGTT) add to the identification of dysglycaemia in CHD patients unknown with diabetes type 2. Without an OGTT a large proportion of patients with new-onset diabetes type 2 or IGT would not have been detected in the EUROASPIRE V survey (Diabetes Care 2020;43: 726-733). Therefore the subgroup considered as normoglycaemic in this study may well include a number of patients with dysglycaemia resulting in misclassification and biased outcomes.

- Another limitation of this study relates to the fact that dysglycaemia was examined at baseline between 2006 and 2017 long before effective drugs became available that may reduce the CV risk(SGLT2, GLP-1RA). Nowadays one should include an OGTT in CR programmes in all patients not known as diabetic and one should personalize the CR programme accordingly using lifestyle changes and appropriate effective and safe pharmacologic therapies.

**Reviewer**	**2**
**Name**	**Formanowicz, Dorota**
**Affiliation**	**Poznan University of Medical Sciences**
**Date**	**14-Jun-2026**
**COI**	**None**

The study addresses an important and clinically relevant question, and benefits from the use of a well-established national registry with a large sample size and long follow-up.

The overall structure is clear, and the results are presented consistently and interpretably. However, several important methodological and interpretative issues should be addressed to strengthen the robustness and clinical relevance of the findings.

My major comments and suggestions are listed below:

1. Residual confounding – lipid-lowering therapy not considered

Although the models adjust for hyperlipidemia, no information is provided on lipid-lowering therapy, including statin use and treatment intensity.

Given that statins are a cornerstone of secondary prevention after myocardial infarction, their omission represents a potential source of residual confounding. Statin treatment may differ across glycaemic categories and independently influences cardiovascular outcomes. Hence, the authors should clarify whether data on statin therapy were available, and, if not, explicitly acknowledge this limitation.

2. Chronic kidney disease (CKD) is not included in adjusted models.

CKD is present in the study population (Table 1), but is not included in the adjusted analyses. Given the well-established association between CKD, inflammation, dysglycaemia, and cardiovascular outcomes, this omission may introduce bias. Evidence suggests that kidney-related mechanisms significantly contribute to cardiovascular mortality risk and should be considered when interpreting such associations (e.g., https://doi.org/10.1038/srep18332).

I recommend: including CKD as a covariate, or performing sensitivity analyses stratified by renal function.

3. Selection bias and survivor bias.

The study population includes only patients who: survived the acute post-MI phase, and

attended a cardiac rehabilitation visit approximately two months after the event.

This introduces:

-selection bias (healthier and more adherent subset),

-survivor bias (exclusion of early mortality and complications).

As a result, the cohort may not fully represent the overall MI population, potentially affecting both risk estimates and generalisability.

4. Glycaemic classification – ambiguity of "possible diabetes."

The category "possible diabetes" fulfills biochemical criteria for diabetes but is analyzed separately from "new-onset diabetes".

This creates conceptual overlap and may introduce: misclassification bias; challenges in clinical interpretation.

A clearer rationale for this classification and its implications is needed.

5. Interpretation of HbA1c versus fasting glucose

The study demonstrates: a near-linear association between fasting glucose and outcomes across all patients; while HbA1c appears predictive mainly in those with established diabetes.

This is an important and potentially clinically relevant finding, but it is underdeveloped in the discussion. The authors are encouraged to expand on: pathophysiological differences (acute vs chronic glycemia); implications for post-MI risk stratification and screening strategies.

6. Clinical interpretation – risk of overstatement

The conclusions suggest that findings support early management of dysglycaemia irrespective of diabetes status.

However, the study is observational; no therapeutic interventions were tested.

The language should be carefully moderated to distinguish between association and causation clearly.

7. Broader interpretation and literature context

The continuous relationship between glycemia and cardiovascular outcomes observed in this study is consistent with prior large-scale analyses demonstrating a graded association between fasting glucose and vascular risk even below diagnostic thresholds (e.g., https://doi.org/10.1016/S0140-6736(10)60484-9).

Additionally, therapeutic modulation of cardiovascular risk (e.g., lipid-lowering strategies) plays a key role in determining outcomes and should be considered when interpreting metabolic risk profiles (e.g., https://doi.org/10.3390/jcm10132876).

My minor comments:

- clarify variability in the timing of the index visit (6–10 weeks) and its potential impact;

- explicitly report the proportion of missing HbA1c data in the main manuscript;

- improve clarity of spline figures (axes, reference values);

- correct minor typographical errors (e.g., “hazard ratio”).

VERSION 1 - AUTHOR RESPONSE

Reviewer 1 comments

1. The study is announced as a ' nationwide cohort study'. One of the strengths is indeed the use of a high-quality national data base to start with. However, only patients who attended a CR program were included (47, 558 out of 227, 859). This selection may well have influenced the results. It is well known that referral to and attendance of CR programs by patients with CHD is limited in many countries. From presentations at international meetings, I recall that in the EUROASPIRE V survey only 74% of eligible patients with CHD were advised to attend a CR program in Sweden and only 65% of those advised did attend CR. Patients who attend CR are in general better off than non-participants. Therefore, the prevalences of pre-diabetes and of new-onset diabetes in this study may well underestimate the real-life prevalences in coronary patients.

   Response: Thank you for your feedback and important comment about patient selection to CR programs. Following sentence has been added to discussion section “*Furthermore, patients without available first CR visit data could not be classified, potentially introducing selection bias and underestimating the prevalence of dysglycaemia after myocardial infarction”* (page 15, paragraph 1).

2. Dysglycaemia was examined in this study using fasting glucose levels (FGL) and HbA1c levels at baseline. It has been shown that in patients with CHD the use of an oral glucose tolerance test (OGTT) adds to the identification of dysglycaemia in CHD patients unknown with diabetes type 2. Without an OGTT a large proportion of patients with new-onset diabetes type 2 or IGT would not have been detected in the EUROASPIRE V survey (Diabetes Care 2020;43: 726-733). Therefore, the subgroup considered as normoglycemic in this study may well include a number of patients with dysglycaemia resulting in misclassification and biased outcomes.

Response: This limitation has been acknowledged Limitation section of the manuscript: “As oral glucose tolerance test (OGTT) data were not available in our study, fasting glucose and HbA1c were used to classify the exposure variable. However, if OGTT had been performed routinely, it might have identified additional cases of previously unrecognised diabetes that would not have been detected by fasting plasma glucose or HbA1c alone” (page 15, paragraph 1).

3. Another limitation of this study relates to the fact that dysglycaemia was examined at baseline between 2006 and 2017 long before effective drugs became available that may reduce the CV risk (SGLT2, GLP-1RA). Nowadays one should include an OGTT in CR programmes in all patients not known as diabetic and one should personalize the CR programme accordingly using lifestyle changes and appropriate effective and safe pharmacologic therapies.

   Response: Thank you for your insightful feedback. In Sweden, both during the study period and at present, the use of OGTT is at the discretion of the treating physician and is therefore not performed routinely. Furthermore, OGTT data are not collected in the SWEDEHEART registry. However, we agree that OGTT may provide additional value in selected cases and have therefore added the following sentence to the Discussion: “As oral glucose tolerance test (OGTT) data were not available in our study, fasting glucose and HbA1c were used to classify the exposure variable. However, if OGTT had been performed routinely, it might have identified additional cases of previously unrecognised diabetes that would not have been detected by fasting plasma glucose or HbA1c alone” (page 15, paragraph 1).

   We agree with the Reviewer that the study period preceded the widespread availability of SGLT2 inhibitors and GLP-1 receptor agonists. This limitation is acknowledged in the manuscript, where we state: “Nevertheless, our findings raise the question of whether novel glucose-lowering agents, such as SGLT2 inhibitors, GLP-1 receptor agonists, oral GLP molecules, or novel incretin combinations, might also play a future role in patients with prediabetes or possible diabetes, as these individuals, according to our study, are at higher risk of developing adverse CV outcome” (page 13, paragraph 3).

Reviewer 2 comments

1. Residual confounding – lipid-lowering therapy not considered
   Although the models adjust for hyperlipidaemia, no information is provided on lipid-lowering therapy, including statin use and treatment intensity.

   Response: Thank you for your feedback. Information on lipid-lowering therapy has now been added to the baseline table (Table 1). At the secondary prevention visit, 96% of all patients were treated with statins, with no meaningful differences across glycaemic categories. Because statin use was nearly universal, it did not contribute relevant variation and could not act as a meaningful confounder.

2. Given that statins are a cornerstone of secondary prevention after myocardial infarction, their omission represents a potential source of residual confounding. Statin treatment may differ across glycaemic categories and independently influences cardiovascular outcomes. Hence, the authors

should clarify whether data on statin therapy were available, and, if not, explicitly acknowledge this limitation.

Response: Information about lipid-lowering therapy has been added to the baseline characteristics table (Table 1). As mentioned above, given the very high proportion of patients receiving statins at baseline (96%), with no meaningful differences across glycaemic categories, we did not believe that including lipid-lowering therapy would contribute meaningfully to the models when designing the study. If the reviewer wishes, we would be willing to repeat the analyses, including lipid-lowering therapy as a confounder in the models.

3. Chronic kidney disease (CKD) is not included in adjusted models. CKD is present in the study population (Table 1) but is not included in the adjusted analyses. Given the well-established association between CKD, inflammation, dysglycaemia, and cardiovascular outcomes, this omission may introduce bias. Evidence suggests that kidney-related mechanisms significantly contribute to cardiovascular mortality risk and should be considered when interpreting such associations (e.g., https://doi.org/10.1038/srep18332). I recommend including CKD as a covariate or performing sensitivity analyses stratified by renal function.

   Response: Thank you for your recommendation. In a sensitivity analysis, we added chronic kidney disease as a covariate to the final model. This addition changed the hazard ratios minimally, with no changes to the findings observed. Following sentence has been added to the Methods section of the manuscript *"In a sensitivity analysis, chronic kidney disease (CKD) was added as an additional covariate to the adjusted model"* (page 7, paragraph 1). Also, we mention this in the Results section of the manuscript: *"In a sensitivity analysis, adding CKD as a covariate had minimal impact on the observed findings (Supplementary Table 2)"* (page 11, paragraph 2).

4. Selection bias and survivor bias. The study population includes only patients who: survived the acute post-MI phase, and attended a cardiac rehabilitation visit approximately two months after the event.
   This introduces:
   - selection bias (healthier and more adherent subset),
   - survivor bias (exclusion of early mortality and complications).
   As a result, the cohort may not fully represent the overall MI population, potentially affecting both risk estimates and generalisability.

   Response: This study utilised data from the cardiac rehabilitation registry within SWEDEHEART, in which information on diabetes status, fasting glucose, and HbA1c is available. Consequently, all patients who attended the cardiac rehabilitation programme were included. As the Reviewer correctly points out, this may introduce both selection and survivor bias. Accordingly, we have added the following limitation: *"As inclusion required participation in a post-MI cardiac rehabilitation visit, selection and survivor bias cannot be excluded"* (page 14, paragraph 3)

5. Glycaemic classification – ambiguity of "possible diabetes". The category "possible diabetes" fulfils biochemical criteria for diabetes but is analysed separately from "new-onset diabetes". This creates conceptual overlap and may introduce misclassification bias; challenges in clinical interpretation. A clearer rationale for this classification and its implications is needed.

Response: As we cannot surely state that these patients have diabetes, they were classified as "possible diabetes". However, agreeing with this ambiguity a sensitivity analysis has been performed combining possible diabetes and new-onset diabetes group. This is now mentioned in the Methods and Results sections of the manuscript: *"A further sensitivity analysis was performed in which patients classified as possible diabetes and new-onset diabetes were combined into a single category"* (page 7, paragraph 1) and *"Furthermore, when possible diabetes and new-onset diabetes were combined into a single category, the results remained unchanged. This combined group had an increased risk of MACE (HR 1.22, 95% CI 1.13–1.31), all-cause mortality (HR 1.49, 95% CI 1.35–1.66), heart failure hospitalisation (HR 1.32, 95% CI 1.20–1.45), cardiovascular mortality (HR 1.21, 95% CI 1.01–1.45), and ischaemic stroke (HR 1.35, 95% CI 1.12–1.61)"* (page 11, paragraph 2).

6. Interpretation of HbA1c versus fasting glucose. The study demonstrates: a near-linear association between fasting glucose and outcomes across all patients, while HbA1c appears predictive mainly in those with established diabetes. This is an important and potentially clinically relevant finding, but it is underdeveloped in the discussion. The authors are encouraged to expand on: pathophysiological differences (acute vs chronic glycemia); implications for post-MI risk stratification and screening strategies.

   Response: Thank you for this suggestion. We have added the following to the Discussion section: *"A near-linear association between fasting glucose and outcomes across all patients, while HbA1c appears predictive mainly in those with established diabetes. This likely reflects different biological processes captured by these biomarkers. Acute hyperglycaemia measure using glucose following MI reflects current metabolic and neurohormonal disturbances, whereas HbA1c reflects cumulative glycaemic exposure and the long-term vascular consequences of chronic hyperglycaemia, which might be particularly relevant in established diabetes"* (page 13, paragraph 1).

7. Clinical interpretation – risk of overstatement. The conclusions suggest that findings support early management of dysglycaemia irrespective of diabetes status. However, the study is observational; no therapeutic interventions were tested. The language should be carefully moderated to distinguish between association and causation clearly.

   Response: The following sentence has been removed from the Conclusion section in the abstract *"…these findings support early management of dysglycaemia…"* and replaced with *"Beyond routine screening, these findings support close follow-up of at-risk patients, irrespective of diabetes status"* (page 3).

8. Broader interpretation and literature context. The continuous relationship between glycemia and cardiovascular outcomes observed in this study is consistent with prior large-scale analyses demonstrating a graded association between fasting glucose and vascular risk even below diagnostic thresholds (e.g., https://doi.org/10.1016/S0140-6736(10)60484-9).

   Response: The following sentence has been added to the Discussion section of the manuscript, where this reference was previously mentioned: *"A near-linear association between fasting glucose and outcomes across all patients, while HbA1c appears predictive mainly in those with established diabetes"* (page 13, paragraph 1) and *"A meta-analysis confirmed the link between*

diabetes and increased risk of CV disease, with near-linear relationship between glucose and coronary heart disease" (page 13, paragraph 2).

9. Additionally, therapeutic modulation of cardiovascular risk (e.g., lipid-lowering strategies) plays a key role in determining outcomes and should be considered when interpreting metabolic risk profiles (e.g., https://doi.org/10.3390/jcm10132876).

   Response: Thank you for this suggestion. The following sentence has been added to the Discussion section of the manuscript: *"In addition, cardiovascular risk following MI is influenced by multiple modifiable factors, including lipid levels and the use of guideline-directed secondary prevention therapies. Although our study focused on glycaemic status, the interpretation of metabolic risk profiles should consider the contribution of comprehensive risk-factor management, including lipid-lowering strategies"* (page 14, paragraph 2).

10. My minor comments: Clarify variability in the timing of the index visit (6–10 weeks) and its potential impact

    Response: The following sentence has been added to the Limitations section of the manuscript: *"The timing of the first cardiac rehabilitation visit varied between 6 and 10 weeks after MI, which may have introduced some variability in metabolic measurements due to differences in recovery phase, lifestyle changes, and early post-MI treatment optimisation. However, any resulting misclassification of glycaemic categories would most likely have been non-differential and therefore expected to attenuate, rather than exaggerate, the observed associations"* (page 15, paragraph 1).

11. Explicitly report the proportion of missing HbA1c data in the main manuscript.

    Response: This has been added *"HbA1c data were available for 13, 994 (30%) of the population"* (page 12, paragraph 1).

12. Improve clarity of spline figures (axes, reference values).

    Response: Figures 1–4 have now been uploaded separately in TIFF format, resulting in improved image clarity.

13. Correct minor typographical errors (e.g., "hazard ratio").

    Response: Minor typographical errors have been corrected.

VERSION 2 - REVIEW

**Reviewer**	**1**
**Name**	**De Backer, Guy**
**Affiliation**	**University of Ghent**
**Date**	**20-Jul-2026**

COI

Thank you for having considered my comments; I do accept your responses and I agree with the adaptations that were made accordingly.

One minor comment: the selection of post-MI patients who attended a cardiac rehab programme is well described, but it is difficult to estimate a possible bias related to this selection; 47558 is a large number but by itself it doesn't not exclude or minimize selection bias. From Fig 1, I estimate that only 21% of all post-MI patients selected in the SWEDEHEART registry during 2006-2017 were included in your study. Is there info on the incidence of MACE in the 180301 post-MI patients who were not included in your study? This could help understanding possible selection bias.

**Reviewer**	**2**
**Name**	**Formanowicz, Dorota**
**Affiliation**	**Poznan University of Medical Sciences**
**Date**	**21-Jul-2026**
**COI**	

Thank you for addressing the previous comments and revising the manuscript.

My main concern remains the interpretation of the findings. The study demonstrates important associations between post-MI dysglycaemia and adverse outcomes, but parts of the Discussion and Conclusions continue to go beyond what can be inferred from an observational study. The manuscript occasionally shifts from risk estimation to potential treatment implications, which I believe should be presented more cautiously.

The distinction between “possible diabetes” and “new-onset diabetes” also deserves further consideration. The observed risks were remarkably similar, yet the clinical interpretation of these two categories remains unclear. As many individuals classified as possible diabetes fulfilled biochemical criteria for diabetes, a more detailed discussion of the clinical relevance of this categorization would be helpful.

Another important finding is the different prognostic behaviour of fasting glucose and HbA1c. This may be one of the most clinically relevant observations of the study and deserves greater emphasis in the discussion.

Finally, the Discussion could be shortened and focused more tightly on the key findings, as some arguments are repeated and dilute the main message.

Overall, this is an interesting and clinically relevant study, but a more restrained interpretation of the results would strengthen the manuscript.

VERSION 2 - AUTHOR RESPONSE

Reviewer 1

1. The selection of post-MI patients who attended a cardiac rehab programme is well described, but it is difficult to estimate a possible bias related to this selection; 47,558 is a large number but by itself it does not exclude or minimize selection bias. From Fig. 1, I estimate that only 21% of all post-MI patients selected in the SWEDEHEART registry during 2006–2017 were included in your study. Is there information on the incidence of MACE in the 180,301 post-MI patients who were not included in your study? This could help understanding possible selection bias.

   Response: We thank the reviewer for this comment. Unfortunately, outcome data for patients who did not attend the first cardiac rehabilitation visit were not available in the current database, and therefore we were unable to compare the incidence of MACE between included and non-included patients. We agree that restricting the study population to patients attending cardiac rehabilitation may introduce selection bias. To acknowledge this limitation more explicitly, we have revised the Discussion and added the following sentence: *"Third, as the study population was limited to patients attending cardiac rehabilitation programme, patients who did not attend could not be evaluated. Consequently, selection and survivor bias cannot be excluded, and the findings may not be fully generalisable to the broader post-MI population."* **(Page 14, paragraph 2)**

Reviewer 2

1. The manuscript occasionally shifts from risk estimation to potential treatment implications, which I believe should be presented more cautiously.

   Response: We thank the reviewer for this important observation. We have revised the conclusions section of the abstract and now further changes have been made to the

Discussion to ensure a more cautious interpretation of our findings (**Page 3**, "conclusions") (**Page 13, Paragraph 1 & 2**)

2. The distinction between "possible diabetes" and "new-onset diabetes" deserves further consideration.

   Response: We appreciate this suggestion and have expanded the Discussion to clarify the clinical relevance of the "possible diabetes" category. We have previously mentioned that individuals fulfilling biochemical criteria for diabetes, despite lacking a formal clinical diagnosis, demonstrated a cardiovascular risk comparable to those with clinically recognised diabetes, but to further extend on this distinction we have revised the following sentence: *"Notably, patients classified as having possible diabetes (elevated glucose and/or HbA1c levels without a clinical diagnosis) exhibited a risk similar to that of new-onset diabetes, suggesting that undiagnosed or early-stage diabetes confers substantial CV risk."* and replaced it with: *"Notably, patients classified as having possible diabetes exhibited a risk similar to that of new-onset diabetes, suggesting that individuals fulfilling biochemical criteria for diabetes may already carry a cardiovascular risk comparable to those with clinically recognised diabetes."* (**Page 12, paragraph 2)**

3. The different prognostic behaviour of fasting glucose and HbA1c deserves greater emphasis.
   Response: In addition to pathophysiology described after the first revision (**Page 12, paragraph 2)**, we now discuss the complementary prognostic roles of fasting glucose and HbA1, and their different associations with cardiovascular outcomes after MI. We have added the following: *"Nevertheless, these findings underscore the prognostic significance of both glucose and HbA1c levels after MI, even when values are within the normal range"* (**Page 12, paragraph 2)**. We have also added the following: *"Our study extends these observations to a post-MI population by distinguishing between various glycaemic states, such as prediabetes, possible diabetes, and new-onset diabetes, and by demonstrating that fasting glucose and HbA1c provide complementary rather than interchangeable prognostic information following MI"* (**Page 13, paragraph 1)**.

4. The Discussion could be shortened and focused more tightly on the key findings.
   Response: Discussion has been revised throughout to improve focus and reduce repetition. We have streamlined the interpretation of the findings, removed redundant statements, and concentrated the discussion on the principal observations of the study (**Page 12 – 15)**.

VERSION 3 - REVIEW

Reviewer 2

Name Formanowicz, Dorota

Affiliation Poznan University of Medical Sciences

Date 26-Aug-2026

COI

The article has been greatly improved. I have no further comments.
